# Highly expressed placental miRNAs control key biological processes in human cancer cell lines

**DOI:** 10.18632/oncotarget.25264

**Published:** 2018-05-04

**Authors:** Daniel Onofre Vidal, Anelisa Ramão, Daniel Guariz Pinheiro, Bruna Rodrigues Muys, Julio Cesar Cetrulo Lorenzi, Cleidson de Pádua Alves, Dalila Luciola Zanette, Greice Andreotti de Molfetta, Geraldo Duarte, Wilson Araújo Silva

**Affiliations:** ^1^ Department of Genetics, Ribeirão Preto Medical School, University of São Paulo, Ribeirão Preto, SP, Brazil; ^2^ Molecular Oncology Research Center, Barretos Cancer Hospital, Barretos, SP, Brazil; ^3^ Center for Cell-Based Therapy (CEPID/FAPESP), National Institute of Science and Technology in Stem Cell and Cell Therapy (INCTC/CNPq), Riberão Preto, SP, Brazil; ^4^ Department of Gynecology and Obstetrics, Ribeirão Preto Medical School, University of São Paulo, Ribeirão Preto, SP, Brazil; ^5^ Center for Medical Genomics (HCFMRP/USP), Ribeirão Preto Medical School, University of São Paulo, Ribeirão Preto, SP, Brazil; ^6^ Center for Integrative Systems Biology (CISBi-NAP/USP), Ribeirão Preto Medical School, University of São Paulo, Ribeirão Preto, SP, Brazil

**Keywords:** human placenta, microRNA, tumorigenesis, miR-451, miR-720

## Abstract

Despite being a healthy tissue, the constituent cells of the placenta, share similar characteristics with tumor cells, such as increased cell growth, migration, and invasion. However, while these processes are stochastic and uncontrolled in cancer cells, in placenta they are precisely controlled. Since miRNAs have been reported to regulate genes that control the molecular mechanisms necessary for the development of both human placenta and cancer, we addressed for miRNAs highly expressed in the placenta that could be involved in tumorigenesis. Here, we assessed the miRNA profile in placenta samples using microarray analysis. The results showed that miR-451 and miR-720, highly expressed placental miRNAs, presented very low or undetectable expression in cancer cell lines compared to the normal placenta and healthy tissues. Additionally, transfection of miR-451 or miR-720 mimics in choriocarcinoma cell line (JEG3) and colorectal adenocarcinoma cell line (HT-29) resulted in impaired cell proliferation, decreased cell migration and invasion and reduced ability of colony formation. These findings provide evidence that placenta may work as an alternative model to identify novel miRNAs involved in pathways controlling tumorigenesis.

## INTRODUCTION

Human placenta is the most specialized transient organ of the pregnancy, which together with the fetal membranes and the amniotic fluid supports the normal growth and development of the embryo [1]. Both the structure and function of the placenta are dictated by the need to maintain fetal growth, keeping the balance between the fetal and maternal systems. Therefore, during pregnancy, the human placenta undergoes a dramatic structural reorganization to functionally synchronize the development of the embryo and the maternal compartment [2]. The development of the placenta is a critical step for the normal growth of the embryo. Trophoblastic cells are responsible for the ability of invasion and proliferation of the placenta [3], properties similar to those associated with the development of cancer. Thereby, despite being a healthy tissue, the constituent cells of the placenta, especially trophoblasts, share similar characteristics with tumor cells, such as increased cell growth, migration, and invasion [3, 4]. However, while these processes are stochastic and uncontrolled in cancer, the cells of the placenta present regulatory mechanisms that precisely control cell growth, migration, and invasion of the uterine tissue [2, 5]. Loss of control of these biological processes may result in the development of choriocarcinoma, a highly malignant disease [6].

MicroRNAs (miRNAs) are small non-coding RNA molecules (19-22 nucleotides) that act in the post-transcriptional regulation of gene expression. In general, this regulation occurs through the binding of a specific miRNA region known as seed (2-8nt in the 5′ region of miRNA) to the 3′ UTR (untranslated region) of the target mRNAs [7, 8]. Several reports have shown that miRNAs play a crucial role in the development of many diseases, including placenta-associated diseases and cancer. Some studies have shown that the development of preeclampsia is associated with altered expression of placental miRNAs [9, 10]. Furthermore, changes in miRNA expression profiles have also been described for several cancers, including breast cancer [11], leukemia [12], prostate cancer [13], among others. Therefore, miRNAs may regulate genes controlling the molecular mechanisms necessary for the development of both human placenta and cancer, such as proliferation, migration, invasion, and apoptosis.

Since the development of placenta shares key molecular mechanisms with the development of cancer, placenta becomes an ideal model to identify novel miRNAs that can be involved in tumorigenesis. For this purpose, we assessed the miRNA expression profile of normal human placenta and evaluated the biological function of highly expressed placental miRNAs in cancer cell lines. From the top five highly expressed placental miRNAs, we demonstrated that miR-451 and miR-720 were highly expressed in normal human placenta and several other normal tissues, but were downregulated in cancer cell lines. Restoration of expression indicates a possible role for miR-451 and miR-720 in controlling proliferation, migration, and invasion at least in choriocarcinoma and colorectal cancer cells.

## RESULTS

### Highly expressed miRNAs in the normal human placenta

miRNA expression analysis was performed in the human placentas ranging from 37 to 40 weeks of gestation (two samples representing each gestational age). RNA quality analysis revealed an average RIN of 6.06 (ranging from 5.1 to 6.7).

**Figure 1:**
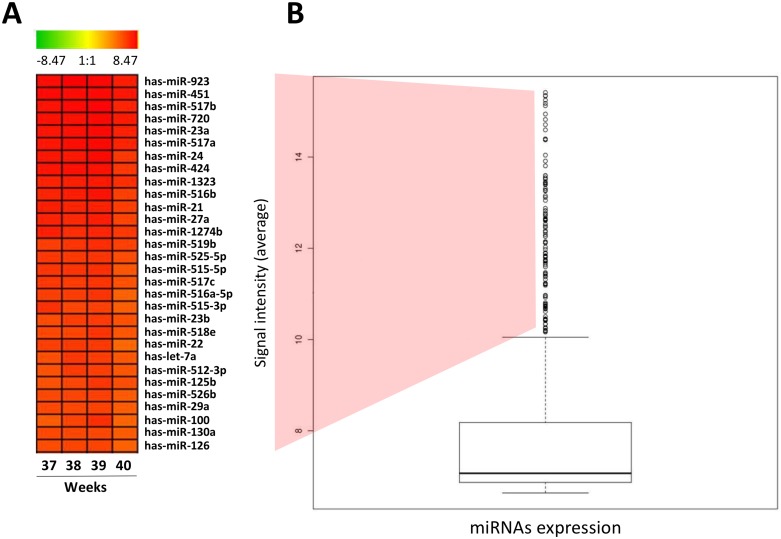
High expressed miRNAs in normal human placenta (**A**) HeatMap representing the top 30 high expressed miRNAs in normal human placenta. (**B**) Boxplot presenting normalized level of expression of the 866 miRNAs evaluated in normal human placenta using microarray miRNA expression profile. The bold line indicates de median of signal intensity of all miRNAs; the top edge represents the 75th percentile and the bottom edge the 25th percentile. Vertical bars indicate the upper and inferior limits and circles represent outlier points/samples (points 1.5 IQR above the 3rd quartile of the boxplot).

Therefore, highly expressed miRNAs in placenta was considered as the ones that presented expression (signal intensity) above the upper limit (outliers: points 1.5 IQR above the 3rd quartile) according the distribution of the microarray expression data in the boxplot. This approach allowed the identification of 235 miRNAs highly expressed in normal human placenta, from which we selected the 30 most highly expressed miRNAs (Figure 1 and Supplementary Table 1).

### miR-451 and miR-720 are highly expressed in normal human placenta and normal tissues but downregulated in cancer cell lines

Among the top-five most highly expressed miRNAs in normal human placenta, we evaluate the expression of miR-451, miR-517a, miR-517b and miR-720 in normal human placenta samples, several normal human tissues and different cancer cell lines using RT-qPCR. miR-923 was excluded from the analysis, because it was reported as a fragment of ribosomal RNA.

**Figure 2:**
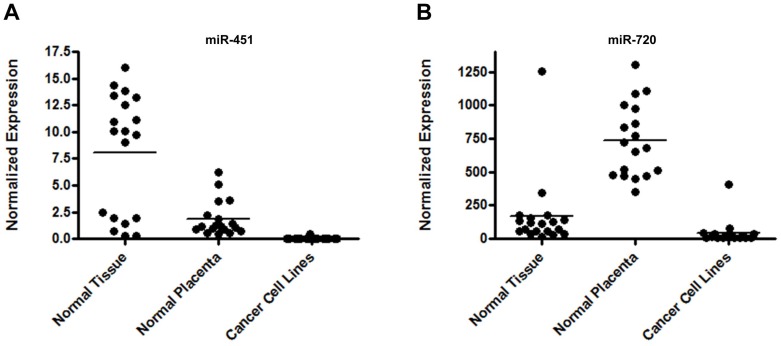
miRNA expression in normal tissues, normal placenta and cancer cell lines Graphic representation of miR-451 (**A**) and miR-720 (**B**) normalized expression evaluated by RT-qPCR. We evaluated the expression of both miRNAs in 20 commercial normal tissues, 19 normal placenta samples and 16 cancer cell lines. A list containing the data for each sample is demonstrated in Supplementary Table 2. Normalized expression was determined using the mathematical model 2^−ΔCq^ (Livak & Schmittgen 2001). The normalization was performed using the endogenous control RNU24.

Our results confirmed miR-451 and miR-720 elevated expression levels in all normal human placenta samples, corroborating the microarray data. Additionally, we demonstrated that the expression of both miRNAs was not restricted to the normal placenta. miR-451 expression was also observed in several human normal tissues, presenting equal or higher expression levels in comparison to the normal placenta (Figure 2A). Similarly, miR-720 expression was also observed in several human normal tissues. However, its expression was lower as compared to the placenta samples (Figure 2B). Interestingly, for both miRNAs, we noticed that their expression in cancer cell lines was very low or undetectable (Figure 2A and 2B; Supplementary Table 2).

For miR-517a and miR-517b, despite the fact that we observed their higher expression in placenta samples, both presented very low or undetectable expression in all the human normal tissues evaluated (data not shown). For this reason, these miRNAs were not considered for the further analysis.

### miR-451 and miR-720 control key biological processes in cancer cell lines

**Figure 3:**
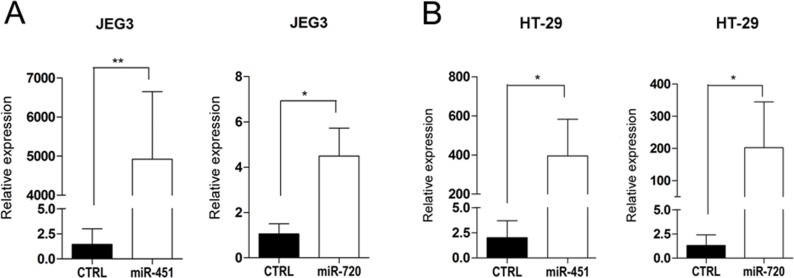
miRNA mimic overexpression in cancer cell lines Graphic representation of miR-451 or miR-720 mimic expression in cancer cell lines JEG3 (**A**) and HT-29 (**B**). Relative expression was evaluated by RT-qPCR using the mathematical model 2^−ΔΔCq^ (Livak & Schmittgen 2001) compared to the control (CTRL). CTRL: parental cancer cell line transfected with irrelevant miRNA mimic (miRIDIAN mimic negative control); miR-451: cancer cell line transfected with miR-451 mimic; miR-720: cancer cell line transfected with miR-720 mimic. Vertical bars represent mean ± the standard deviation of independent triplicates. Mann-Whitney statistical test; ^*^p < 0.05, ^**^p < 0.01.

miR-451 and miR-720 were expressed in normal tissues, including placenta, but downregulated in cancer cell lines as compared to normal samples, suggesting that both miRNAs could be important to the maintenance of the normal phenotype in placenta cells and normal tissues. Therefore, we assessed whether these miRNAs could be involved in the regulation of cellular biological processes critical for tumor development. Using miRNA mimics, we overexpressed miR-451 or miR-720 (Figure 3A and 3B) in a cancer cell line derived from choriocarcinoma (JEG3) and a colon adenocarcinoma cell line (HT-29), both expressing low levels of those miRNAs (data not shown).

**Figure 4:**
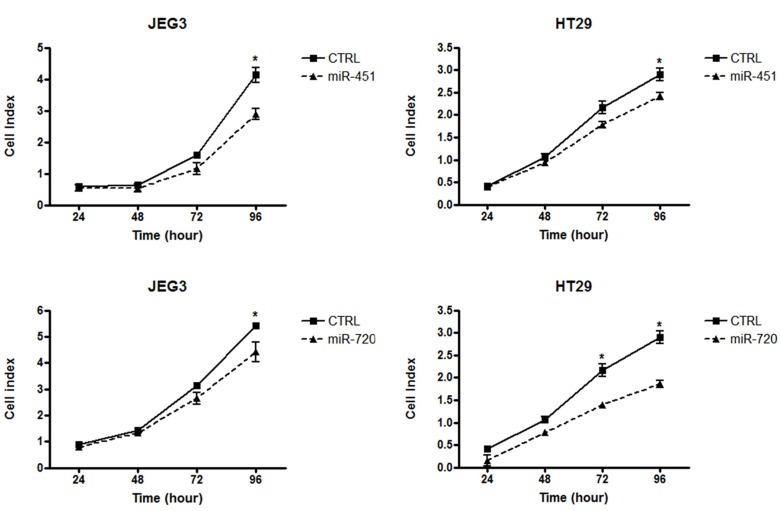
Cell proliferation index Graphical representation of cell index for cancer cell lines JEG3 and HT-29. Cell proliferation assay was performed in xCELLigence system (Roche). Cell index value was acquired at 24, 48, 72 and 96 h. CTRL: parental cancer cell line transfected with the irrelevant miRNA mimic (miRIDIAN mimic negative control); miR-451: cancer cell line transfected with miR-451 mimic; miR-720: cancer cell line transfected with miR-720 mimic. Each point represent mean ± the standard deviation of independent triplicates. Mann-Whitney statistical test; ^*^p < 0.05.

**Figure 5:**
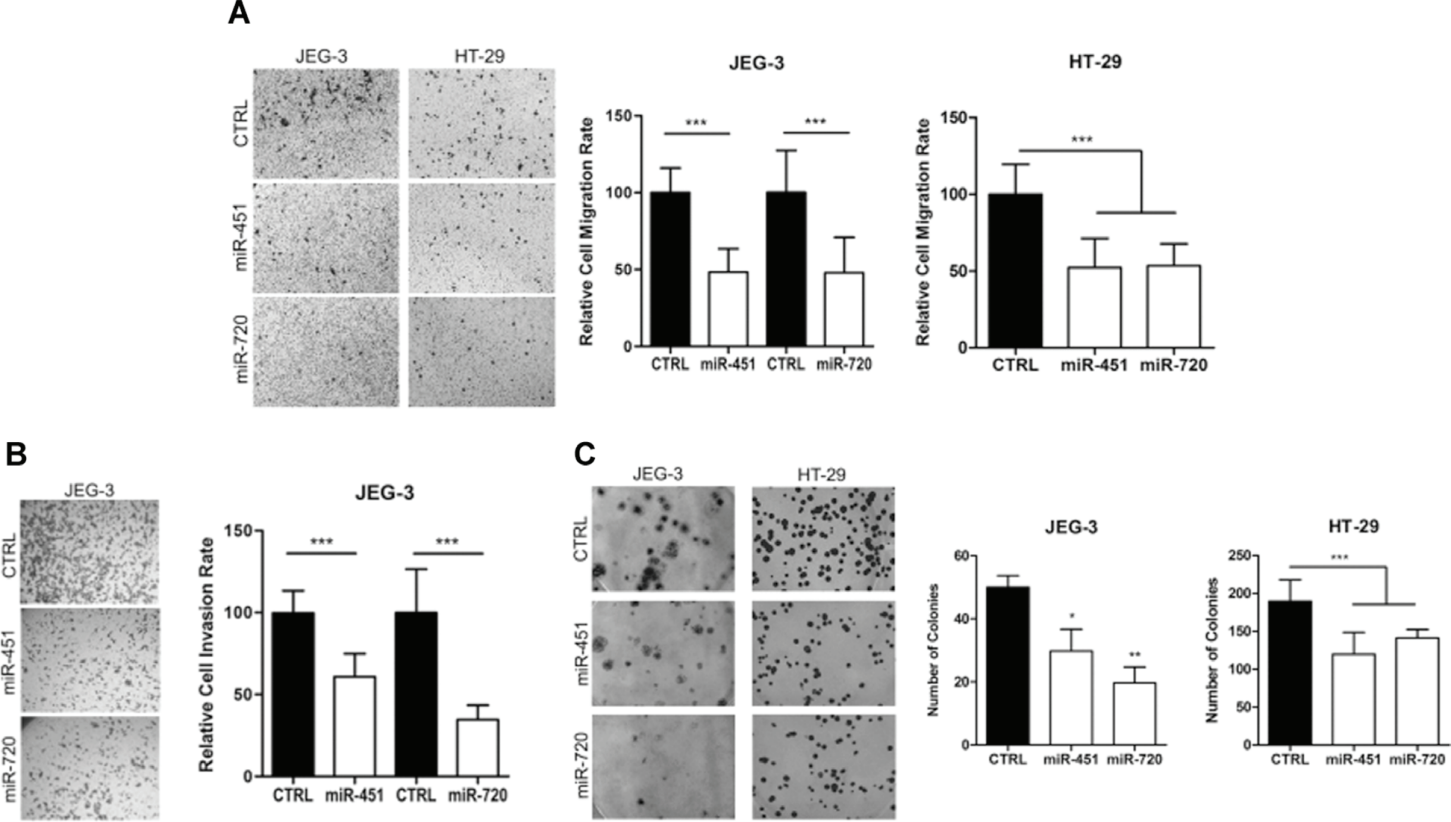
Cell migration, invasion and colony formation ability Cell migration, invasion and colony formation ability after miR-451 or miR-720 mimic transfection. (**A**) Cell migration rate; (**B**) Cell invasion rate; (**C**) Colony formation ability. Cells were allowed to migrate/invade for 24 h at 37° C and 5% CO_2_ and colony formation was evaluated after 12 days. CTRL: parental cancer cell line transfected with the irrelevant miRNA mimic (miRIDIAN mimic negative control); miR-451: cancer cell line transfected with miR-451 mimic; miR-720: cancer cell line transfected with miR-720 mimic. Vertical bars represent mean ± the standard deviation of independent triplicates. Mann-Whitney statistical test (migration and invasion); One-Way ANOVA statistical test (colony formation); ^*^P < 0.05; ^**^P < 0.01; ^***^P < 0.001.

Our data demonstrated that miR-451 or miR-720 ectopic expression impaired cell proliferation in both JEG3 and HT-29 cancer cell lines (Figure 4). Additionally, we observed that overexpression of miR-451 or miR-720 dramatically decreased cell migration in both cell lines (Figure 5A). JEG3 cells also had their invasion ability impaired upon overexpressed miR-451 or miR-720 (Figure 5B). On the other hand, HT-29 cells did not show invasiveness ability, even when we used more cells/well or maintained the experimental conditions for longer periods (data not shown). Furthermore, colony formation assay demonstrated that miR-451 or miR-720 overexpression significantly reduced the ability of both JEG3 and HT-29 cells to establish colonies after twelve days of culturing (Figure 5C).

## DISCUSSION

Some miRNAs have been described to be exclusively expressed in human placenta [14, 15]. Most of them are identified in maternal plasma during pregnancy [16]. Such observations suggest that miRNAs may play an important role in maternal-fetal communication, possibly promoting maternal adaptation to pregnancy [15, 17]. Moreover, miRNA differential expression in maternal plasma has been used as a marker to predict complications during pregnancy, such as preeclampsia [18, 19].

The decreased expression of placental miRNAs has also been described, contributing to the regulation of tumor invasion [20], cell proliferation, migration and differentiation [21]. Recently, our group demonstrated that the restoration of the expression of placenta-enriched long intergenic non-coding RNAs (lincRNAs) was associated with a decrease in cell migration and invasion of the JEG-3 cell line [22].

In this report, we demonstrated that miR-451 and miR-720 highly expressed placental miRNAs, presented very low or undetectable expression in cancer cell lines when compared to the normal placenta and other normal tissues. Additionally, ectopic expression of miR-451 or miR-720 in choriocarcinoma cell line (JEG3) or colon adenocarcinoma cell line (HT-29) resulted in impaired cell proliferation, decreased cell migration and reduced ability of colony formation in both cells lines. Also, it was associated with a reduction in the invasion ability of JEG3 cancer cells.

Scientific literature regarding miR-451 and miR-720 expression in human placenta is scarce. However, miR-451 and miR-720 elevated expression in human placenta samples has already been demonstrated in previous studies [15]. Additionally, the differential expression of miR-451 was related with placenta undergoing hypoxic conditions [23]. MiR-451 was suggested as a tumor suppressor as it has been associated with the regulation of several biological processes, including cell proliferation, migration, invasion and treatment response in glioblastoma [24, 25], colorectal carcinoma [26, 27] and lung cancer cell lines [28, 29]. MiR-451 low expression was also associated with cell survival and resistance to hormone therapy in patients with breast carcinoma [30, 31] and with the progression and worse prognosis in osteosarcoma [32, 33].

Although few reports are available, data have been shown to be controversial regarding miR-720 expression in several tumor types. In accordance with our findings, some have reported miR-720 among miRNAs with lower expression levels in renal carcinoma [34], gastric lymphomas [35], pancreatic carcinoma [36] and esophageal carcinoma [37]. Also, miR-720 low expression was observed in lung carcinoma patients with poorer survival [38] while restored expression was associated with inhibition of tumor invasion and migration of breast cancer [39]. Nonetheless, miR-720 has also been demonstrated with increased expression in myelodysplastic syndrome [40], multiple myeloma [41], colorectal carcinoma [42] and melanoma [43]. Our data, together with these observations suggest that the role of miR-720, as an oncogene or tumor suppressor, depends on the cellular context, which has also been demonstrated for other miRNAs [44].

In summary, our results highlight the human placenta as an applicable model for the study of tumorigenesis. Furthermore, we demonstrated that the evaluation of highly expressed miRNAs in human placenta is a powerful strategy for the identification of miRNAs involved in the regulation of important biological processes in cancer cells. Together, our data indicate a possible role for miR-451 and miR-720 in controlling proliferation, migration, and invasion at least in choriocarcinoma and colorectal cancer cells. Future studies are necessary to gain further mechanistic insights into miR-451 and miR-720 role in tumorigenesis and assess if these findings can be translated to clinical application.

## MATERIALS AND METHODS

### Human normal placenta samples

A total of 21 normal human placenta samples (35 to 40 weeks) were collected at birth by cesarean or vaginal delivery from healthy women (age range from 21 to 33, mean: 27,7; years). We excluded placentas from women presenting hypertension, preeclampsia, placenta previa, preterm labor, diabetes, autoimmune disease or infections (HPV, HIV, and others). The samples were collected at the obstetric center of the Clinics Hospital of the Ribeirão Preto Medical School – University of São Paulo (HC-FMRP/USP), and at the Woman Health Reference Center of Ribeirão Preto (CRSM-Mater).

The study was conducted following the national and institutional ethical policies and was previously approved by the Institutional Ethical Committee from both institutions (protocol # 9411/2010 for HC-FMRPUSP; and 009/2010 for CRSM-Mater). All the pregnant women included in the study agreed to participate by signing a specific consent form.

### Human cancer cell lines and normal tissues

We evaluated a total of 16 human cancer cell lines comprising nine different human tissues: central nervous system (T98G), colorectal cancer (Caco-2, DLD-1 and HT29), breast cancer (MCF7, SKBR3 and HCC1954), head and neck cancer (FaDu and UM-SCC-14), liver cancer (HEPG2), leukemia (NB4, Jurkat and K562), lymphoma (U937), prostate cancer (LnCap) and choriocarcinoma (JEG3). The cancer cell lines were maintained in appropriated media, RPMI or MEM, containing 10% of Fetal Bovine Serum (FBS), incubated at 37° C and 5% CO_2_ and tested regularly for mycoplasma contamination using the MycoAlert^™^ PLUS Mycoplasma Detection Kit (Lonza. Allendale, NJ, USA). Authentication of cell lines was performed by short tandem repeat (STR) DNA typing according to the International Reference Standard for Authentication of Human Cell Lines, as reported by Dirks *et al*. [45]. Genotyping confirmed the identity of all cell lines.

We also used a commercial panel of total RNAs extracted from 20 different normal human tissues, FirstChoice Human Total RNA Panel Survey (Ambion, Austin, TX, USA).

### RNA extraction and quality assessment

The total RNA from normal placenta samples and human cancer cell lines was isolated using TRIZOL (Invitrogen, Grand Island, NY, USA), according to the manufacturer's instructions [15]. RNA was quantified using Nanodrop 1000 Spectrophotometer (ThermoFisher Scientific, Waltham, MA USA). RNA quality was assessed using the Eukaryote RNA Total Pico kit (Agilent Technologies, Santa Clara, CA, USA) in Bioanalyzer 2100 (Agilent Technologies, Santa Clara, CA, USA), according to manufacturer's instructions.

### miRNA expression in normal placenta samples

miRNA expression profile of normal placenta was performed with Human miRNA Microarray Version 3 platform (cat# G4470C, Agilent Technologies, Santa Clara, CA, USA) and miRNA Complete Labeling and Hyb Kit (Agilent Technologies, Santa Clara, CA, USA), according to manufacturer's instructions. The platform contains specific probes for the expression analysis of 866 human miRNAs described in public databases (miRBase release 12.0) [46]. Microarray slides were scanned using the High-Resolution Microarray Scanner (Agilent Technologies, Santa Clara, CA, USA). Captured images were processed, and raw data were extracted by the Agilent Feature Extraction Software v.8.5 (Agilent Technologies, Santa Clara, CA, USA).

### Microarray analysis

Raw data files from miRNA expression for each of the human normal placenta samples were loaded and analyzed using R software (http://www.r-project.org/). Initially, we extracted the signal background of the slides and then filtered the probes that did not show fluorescence signal intensity. Data normalization was performed using the 90th percentile and subsequently transformed to a logarithmic scale. Highly expressed miRNAs in placenta was considered as the ones that presented expression (signal intensity) above the upper limit (outliers: points 1.5 IQR above the 3rd quartile) according the distribution of the microarray expression data in boxplot graphical tool. To the analysis and data visualization (charts and graphs), we used packages (limma and AgiMicroRNA) available at the public database Bioconductor (http://www.bioconductor.org).

### miRNA expression validation

miRNA expression validation was performed for normal placenta and cancer cells lines using TaqMan MicroRNA Assays (Applied Biosystems, Foster City, CA, USA). Initially, 20 ng of total RNA was submitted to miRNA-specific cDNA synthesis using High Capacity Reverse Transcription kit (Applied Biosystems, Foster City, CA, USA) according to the following conditions: 1× enzyme buffer, 1 mM dNTPs, 1 μl of specific stem-loop primer, 4 U RNAse inhibitor, 50 U Multiscribe Reverse Transcriptase in a final volume of 15 μl. Reaction conditions followed according to the manufacturer's instructions.

cDNA was used as template for the RT-qPCR using the TaqMan Fast Universal PCR Master Mix (Applied Biosystems, Foster City, CA, USA) in the following conditions: 1X Master Mix, 0.5 μl of specific primer pair and probe, and 4 μl of cDNA (diluted in a ratio 1:4) to a final volume of 10 μl. All the reactions were performed in duplicates using standard reaction conditions in ABI Prism 7500 Sequence Detection System (Applied Biosystems, Foster City, CA, USA).

miRNA endogenous controls (RNU24, RNU44 and RNU48) were also evaluated in all samples. Expression normalization was performed using RNU24, which presented the lowest expression variation (<1Ct) among all samples. Normalized expression levels of miRNAs were calculated using the mathematical model proposed by Livak and Schmittgen (2001) [47].

### miRNA ectopic expression and functional assays

#### miRNA mimics transfection

The ectopic expression of miR-451 or miR-720 was performed in cancer cell lines JEG3 (choriocarcinoma) and HT-29 (colon adenocarcinoma). miRNA overexpression was performed using miRIDIAN mimics assay (Dharmacon, Lafayette, CO, USA). Cell line transfections were carried out with DOTAP (Roche Applied Science, Indianapolis, IN, USA) following the protocol described by Martino *et al.* (2009) [48].

### Cell proliferation assay

Cell proliferation assays were performed using the xCELLigence System (Roche Applied Science, Indianapolis, IN, USA), which allows the real time monitoring of cell proliferation. Briefly, 10,000 cells of JEG3 or HT-29 were transfected in the E-plate 16 wells (Roche Applied Science, Indianapolis, IN, USA) to a final concentration of 50 nM of miRIDIAN mimics (Dharmacon, Lafayette, CO, USA) in 200 μl of MEM containing 10% FBS. Culture medium was replaced every 24 hours. Cell index value was acquired at 24, 48, 72 and 96 h. All experiment conditions were done in triplicates.

### Migration and invasion assays

Cell motility and invasion ability were evaluated in 24-well transwell inserts (8 μm pores – Greiner Bio-One, Monroe, NC, USA) or 24-well transwell inserts containing Matrigel (Corning, New York, NY, USA), respectively. JEG3 and HT-29 cells (1 × 10^5^ cells/200 μL serum free medium) were seeded in the upper chambers of the transwell inserts 48 h after transfection with mimics for miR-451 or miR-720 or negative control. Lower chambers were filled with 500 μL of medium added 10% FBS. JEG3 cells were allowed to migrate/invade for 24 h at 37° C and 5% CO_2_. HT-29 cells were allowed to migrate for 24h and invade for 48 h at 37° C and 5% CO_2_. Then, cells from the upper compartment were removed with a cotton swab and insert membrane were fixed with 4% formaldehyde (in PBS) and stained with 0.5% crystal violet. The number of cells was manually counted using ImageJ software. All experiment conditions were performed in triplicates.

### Colony formation assay

Cells overexpressing miR-451 or miR-720 for 48h were harvested using TrypLE Express enzyme (ThermoFisher Scientific, Waltham, MA USA) and plated sparsely into 6-well plates (500 cells/well). After 12 days, cells were washed with PBS 1×, fixed in 4% formaldehyde (in PBS) and stained with 0.5% crystal violet. The number of colonies was counted using ImageJ software. All experiment conditions were performed in triplicates.

## SUPPLEMENTARY MATERIALS AND TABLES




