# Supplementary material for: Highly expressed placental miRNAs control key biological processes in human cancer cell lines
Source: Oncotarget. 2018 May 4;9(34):23554–63. doi: 10.18632/oncotarget.25264 (PMC5955126; doi:10.18632/oncotarget.25264)
Supplement: Supplementary file 1 [file oncotarget-09-23554-s001.pdf]

Highly expressed placental miRNAs control key biological processes in human cancer cell lines

SUPPLEMENTARY MATERIALS

Supplementary Table 1: The 30 most highly expressed miRNAs in normal human placenta samples of the third trimester of pregnancy

miRNAs	37 weeks*	38 weeks*	39 weeks*	40 weeks*	Average**
hsa-miR-451	15.554	15.489	15.080	15.650	15.443
hsa-miR-923	15.745	15.218	15.650	14.990	15.401
hsa-miR-517b	15.121	15.489	15.542	15.427	15.395
hsa-miR-720	15.381	15.149	15.181	15.073	15.196
hsa-miR-517a	14.888	15.542	15.039	15.190	15.165
hsa-miR-23a	15.109	15.149	15.307	14.871	15.109
hsa-miR-424	15.264	14.970	15.015	15.156	15.101
hsa-miR-24	15.238	14.702	15.266	14.758	14.991
hsa-miR-21	14.419	15.113	14.301	15.156	14.747
hsa-miR-516b	14.691	14.388	14.970	14.464	14.628
hsa-miR-1323	14.332	14.749	14.702	14.340	14.531
hsa-miR-1274b	14.512	14.739	14.305	14.493	14.512
hsa-miR-27a	14.492	14.332	14.558	14.465	14.462
hsa-miR-515-5p	13.974	13.951	13.873	14.087	13.971
hsa-miR-517c	13.540	14.030	13.507	14.219	13.824
hsa-miR-519d	13.690	13.643	14.087	13.665	13.771
hsa-miR-525-5p	13.825	13.974	13.715	13.390	13.726
hsa-miR-515-3p	14.081	13.944	12.972	13.513	13.627
hsa-miR-516a-5p	13.837	13.209	13.837	13.401	13.571
hsa-let-7a	12.999	13.757	13.744	13.728	13.557
hsa-miR-22	13.743	13.619	13.797	12.974	13.533
hsa-miR-23b	13.192	13.431	13.677	13.507	13.452
hsa-miR-126	13.153	13.431	13.304	13.662	13.388
hsa-miR-518e	13.366	13.259	13.629	13.208	13.365
hsa-miR-29a	13.555	13.119	13.245	13.496	13.354
hsa-miR-512-3p	13.447	12.900	13.544	13.430	13.330
hsa-miR-130a	13.431	13.131	13.370	13.153	13.271
hsa-miR-125b	12.899	13.300	13.743	13.056	13.249
hsa-miR-30b	12.912	13.476	13.347	13.146	13.220
hsa-miR-26a	13.321	13.366	12.917	13.274	13.220
hsa-miR-1225-5p	10.936	12.868	12.111	12.520	12.109

**Log* of the normalized average expression of the miRNAs in the two placenta samples for each gestational weeks used in the microarrays experiments.
***Log* of the normalized average expression of the miRNAs for all the placenta samples used in the microarrays experiments.

Supplementary Table 2: Normalized expression of miR-451 and miR-720 in the 16 cancer cell lines, 20 commercial normal tissues samples and 19 normal placenta samples. See supplementary_Table_2
