# Supplementary material for: Highly expressed placental miRNAs control key biological processes in human cancer cell lines
Source: Oncotarget. 2018 May 4;9(34):23554–63. doi: 10.18632/oncotarget.25264 (PMC5955126; doi:10.18632/oncotarget.25264)
Supplement: Supplementary file 2 [file oncotarget-09-23554-s002.docx]

**Supplementary Table 2:** **Normalized Expression of miR-451 and miR-720 in the 16 cancer cell lines, 20 commercial normal tissues samples and 19 normal placenta samples**

| **miRNA expression in normal tissue** | | | | |
| --- | --- | --- | --- | --- |
| **miR-451** | |  | **miR-720** | |
| **Sample** | **2^-ΔCt^** |  | **Sample** | **2^-ΔCt^** |
| Placenta | 48.04121436 |  | Placenta | 1513.522073 |
| Kidney | 15.99217524 |  | Skeletal Muscle | 1256.660858 |
| Adipose | 14.37645797 |  | Liver | 342.2455783 |
| Ovary | 13.81049403 |  | Prostate | 174.0948119 |
| Prostate | 13.38589269 |  | Kidney | 173.9590669 |
| Bladder | 13.2696699 |  | Cervix | 153.8882682 |
| Trachea | 12.5213354 |  | Trachea | 138.5672802 |
| Testis | 11.13132142 |  | Esophagus | 135.8692178 |
| Lung | 10.92247794 |  | Spleen | 125.401544 |
| Skeletal Muscle | 10.09011906 |  | Bladder | 121.7134908 |
| Brain | 10.04254152 |  | Heart | 110.8528037 |
| Spleen | 9.722274507 |  | Brain | 73.90536894 |
| Esophagus | 8.992249706 |  | Testis | 72.43109942 |
| Thyroid | 2.433626483 |  | Small Intestine | 58.58538686 |
| Colon | 1.968999532 |  | Adipose | 55.72802483 |
| Cervix | 1.960427811 |  | Lung | 53.8471909 |
| Liver | 1.383587904 |  | Colon | 37.07247319 |
| Thymus | 0.716543846 |  | Thyroid | 35.08670474 |
| Heart | 0.299233208 |  | Thymus | 27.63289516 |
| Small Intestine | 0.282735052 |  | Ovary | 17.54233188 |

| **miRNA expression in normal placenta** | | | | | | |
| --- | --- | --- | --- | --- | --- | --- |
| **miR-451** | | |  | **miR-720** | | |
| **Sample** | **Week of gestation** | **2^-ΔCt^** |  | **Sample** | **Week of gestation** | **2^-ΔCt^** |
| Sample 20 | 35w | 6.241807907 |  | Sample 11 | 40w | 3574.146051 |
| Sample 1 | 40w | 5.089522699 |  | Sample 9 | 40w | 1302.266849 |
| Sample 3 | 37w | 3.643781762 |  | Sample 7 | 39w | 1107.862821 |
| Sample 4 | 37w | 3.481404058 |  | Sample 3 | 37w | 1089.514672 |
| Sample 2 | 37w | 2.188785788 |  | Sample 14 | 40w | 1002.22473 |
| Sample 19 | 39w | 1.83791357 |  | Sample 1 | 40w | 976.9999898 |
| Sample 9 | 40w | 1.45597204 |  | Sample 2 | 37w | 863.841857 |
| Sample 10 | 40w | 1.238978108 |  | Sample 20 | 35w | 832.7481596 |
| Sample 6 | 38w | 1.168664763 |  | Sample 12 | 40w | 771.9488521 |
| Sample 5 | 38w | 1.167864695 |  | Sample 10 | 40w | 723.689748 |
| Sample 16 | 39w | 1.035332813 |  | Sample 18 | 37w | 677.0811246 |
| Sample 7 | 39w | 1.006420682 |  | Sample 8 | 38w | 652.9091843 |
| Sample 12 | 40w | 0.911412843 |  | Sample 4 | 37w | 517.5513655 |
| Sample 13 | 38w | 0.851324057 |  | Sample 6 | 38w | 511.5290932 |
| Sample 18 | 37w | 0.7133745 |  | Sample 13 | 38w | 479.2752963 |
| Sample 14 | 40w | 0.571961046 |  | Sample 16 | 39w | 471.1974718 |
| Sample 8 | 38w | 0.519559469 |  | Sample 5 | 38w | 469.1809247 |
| Sample 17 | 38w | 0.451163643 |  | Sample 17 | 38w | 451.9802876 |
| Sample 11 | 40w | 0.207160493 |  | Sample 19 | 39w | 349.2809368 |

| **miRNA expression in cancer cell lines** | | | | | | |
| --- | --- | --- | --- | --- | --- | --- |
| **miR-451** | | |  | **miR-720** | | |
| **Cell Line** | **Cancer type** | **2^-ΔCt^** |  | **Cell Line** | **Cancer type** | **2^-ΔCt^** |
| T98G | glioblastoma | 0.47306325 |  | NB4 | myeloid leukemia | 409.861116 |
| JEG3 | choriocarcinoma | 0.14557488 |  | Jurkat | lymphoid leukemia | 74.8252051 |
| SK-BR-3 | breast cancer | 0.0050552 |  | DLD1 | colorectal cancer | 44.2328051 |
| MCF7 | breast cancer | 0.00499232 |  | K562 | myeloid leukemia | 33.2326883 |
| K562 | myeloid leukemia | 0.00074384 |  | HT29 | colorectal cancer | 18.3487624 |
| NB4 | myeloid leukemia | 0.00036367 |  | HEPG2 | hepatocellular carcer | 17.0545061 |
| DLD1 | colorectal cancer | 0.00034614 |  | T98G | glioblastoma | 14.0774424 |
| HEPG2 | hepatocellular cancer | 0.00029761 |  | U937 | lymphoma | 11.9391572 |
| Jurkat | lymphoid leukemia | 0.00021759 |  | LnCap | prostate cancer | 10.2046062 |
| U937 | lymphoma | 0.00020918 |  | Caco-2 | colorectal cancer | 9.16272639 |
| FaDu | pharinx cancer | 0.00013543 |  | UM-SCC-14 | head and neck cancer | 8.77108477 |
| HT29 | colorectal cancer | 9.5131E-05 |  | FaDu | pharinx cancer | 8.66403953 |
| LnCap | prostate cancer | 9.1748E-05 |  | HCC1954 | breast cancer | 8.0030678 |
| HCC1954 | breast cancer | 7.5625E-05 |  | JEG3 | choriocarcinoma | 7.37274648 |
| UM-SCC-14 | heand and neck cancer | 3.114E-05 |  | SK-BR-3 | breast cancer | 7.34990499 |
| Caco-2 | colorectal cancer | 2.6845E-05 |  | MCF7 | breast cancer | 7.34515486 |
